# Impact-Sliding Tribology Behavior of TC17 Alloy Treated by Laser Shock Peening

**DOI:** 10.3390/ma11071229

**Published:** 2018-07-17

**Authors:** Meigui Yin, Wenjian Wang, Weifeng He, Zhenbing Cai

**Affiliations:** 1 Tribology Research Institute, Key Lab of Advanced Technologies of Materials, Southwest Jiaotong University, Chengdu 610031, China; yinmeigui1618@my.swjtu.edu.cn (M.Y.); wwj527@163.com (W.W.); 2 Science and Technology on Plasma Dynamics Laboratory, Air Force Engineering University, Xi’an 710038, China; hehe_coco@163.com

**Keywords:** Impact-sliding wear, titanium alloy, laser shock peening, wear rate

## Abstract

Outer particle collision with certain dynamic objects is not a pure impact wear behavior; it is typically accompanied by sliding wear phenomena. This study is aimed at investigating the impact-sliding wear performance of three different TC17 titanium alloys. One was untreated, and the other two were subjected to laser shock peening (LSP) by 5 and 7 J pulse energy, respectively. The wear test was performed on a novel impact-sliding wear testing rig, which can realize multiple impact-sliding motions by changing motion parameters in the x and z directions. Present results showed that wear resistance of both treated samples improved compared with the untreated alloy. Given the increase in wear cycles, increment in wear rate of the untreated sample was constantly higher than those of the treated samples. All results can be attributed to the increase in surface hardness of the material and residual compressive stress, which was also introduced after LSP.

## 1. Introduction

Considering the rapid development of the aviation industry, more types of high performance titanium alloys have been widely used in the aviation manufacturing field. These materials commonly exhibit excellent properties, such as high strength to weight ratio, excellent toughness, and outstanding corrosion resistance [1,2]. TC17 titanium alloy has been widely used to manufacture aero-engine fans, compressor disks, jet-engine blades, and other important parts of advanced aircrafts [3,4]. However, titanium alloys usually exhibit poor wear resistance and low surface hardness, due to their structural characteristics, which always limits their wide application [5,6].

In recent years, researchers have proposed several surface treatment technologies to improve mechanical properties of titanium alloys. Some coatings, such as carbide, nitride oxide, and other compounds, have been commonly used to improve superficial properties of materials to protect substrates against material degradation and failure [7,8]. Liu et al. [9,10,11] studied the microstructural characteristics of TC17 after shot peening, which was achieved by projecting some small particles on the surface of samples to form a strengthened layer with a certain thickness; some heat treatment techniques are also applied to improve friction and wear properties of titanium alloys [12,13]. Qiao et al. [14,15] investigated laser peening on TC17 titanium alloy microstructure and properties. Laser shock peening (LSP) is a surface treatment method that can improve fatigue, wear, and corrosion properties of materials mainly by increasing residual compressive stresses and refining the crystalline structure. Compared to other methods, LSP exhibits unique advantages, such as non-contact, non-pollution, and non-thermal effects. Since laser size can be freely changed, this method can handle some areas that cannot be processed by some other traditional methods [16]. 

In actual wear conditions, the motion states of friction pairs are complex and diverse. During the sliding wear process, the impactor always moves tangentially relative to the wear specimen [17], the impact fretting wear implies that fixed sample is subjected to shock by a dynamic impactor [18,19]. Impact-sliding fretting wear indicates that tangential friction occurs simultaneously when mechanical components are subjected to impact [20,21]. Working aero-engine fan blades are easily attacked by solid particles suspended in air, resulting in wear which increases the surface roughness of the blade, causing permanent loss of performance [22]. Sato et al. [23] observed that the wear volume resulting from impact-sliding wear was more significant than that from pure impact or pure sliding fretting wear. 

This study investigated the wear behaviors of three different TC17 titanium alloys with a self-developed impact-sliding fretting wear test rig. Changes in microstructures and mechanical properties of two peened samples were also analyzed.

## 2. Experimental Details

### 2.1. Materials

TC17 titanium alloy primarily comprises hexagonal close-packed α phases with several body centered cubic β phases. The chemical composition (wt.%) of TC17 alloy includes 4.1% Al, 2.9% Sn, 2.0% Zr, 4.3% Mo, 4.2% Cr, and balanced Ti. Before LSP, all samples were polished with a Sic paper and a polishing cloth, then, the surface of samples was washed with alcohol in an ultrasonic bath and dried using compressed air. 

### 2.2. LSP

**Figure 1:**
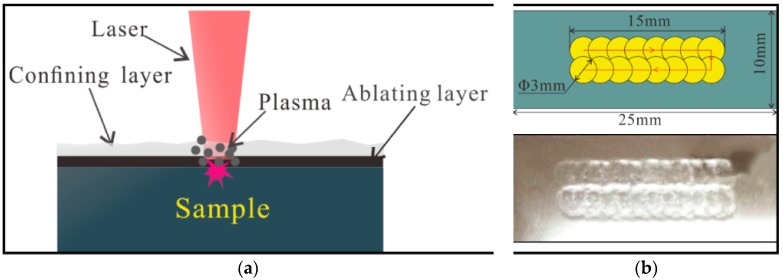
Principle of laser shock peening (LSP). (**a**) Laser shock peening; (**b**) LSP region.

Figure 1 shows the schematic diagram of LSP. During LSP, a high-power laser beam is irradiated on the ablating layer which always served to increase absorbance of laser energy and avoid overheating on the sample’s surface. Plasmas also form on the irradiated surface. The confining layer is applied to prevent the plasma from expanding away from the surface primarily for obtaining a high-magnitude shock wave, which is then transmitted into the sample. This work used a laser with a wavelength of 1064 nm, spot diameter of 3 mm and pulse duration of 10 ns. Both treated samples were peened twice by laser pulse energy of 5 and 7 J, respectively.

### 2.3. Impact-Sliding Fretting Wear Test Method

**Figure 2:**
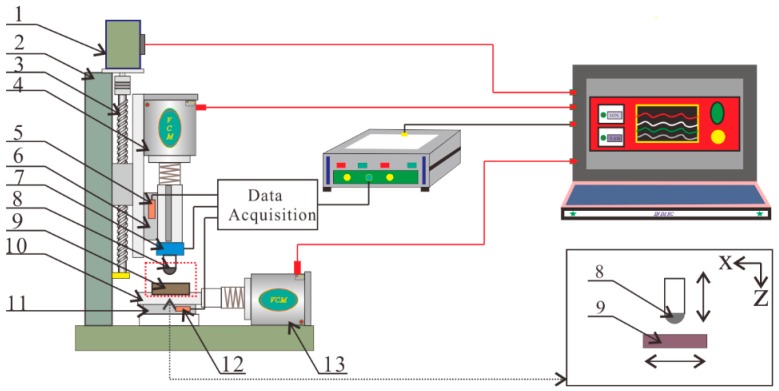
Schematic diagram of the impact-sliding fretting wear test rig. **1**. Servo motor, **2**. Frame, **3**. Lead screw, **4**. Z-direction voice coil motor, **5**. Z-direction displacement transducer, **6**. Z-direction liner slider, **7**. 2-D force sensor, **8**. Impact head, **9**. Test specimen, **10**. Sample clamp, **11**. X-direction liner slider, **12**. X-direction displacement transducer **13**. X-direction voice coil motor.

This research was performed on a homemade impact-sliding fretting wear test rig. Figure 2 illustrates the mechanical structure of this rig. The voice coil motor (4) fixed at the z direction was used to drive the impact head (8), whereas the test sample (9) was driven by another voice coil motor (13) fixed on the x direction. Both motors can realize different parameters of sinusoidal motion for the impact head and test samples. The normal impacting force and horizontal tangential force formed during the wear process, which were then gathered by a 2-D force sensor (7). Two displacement transducers (5, 12) were employed to record movement of impact head and test sample throughout the wear process. The servo motor (1) was employed to attain the required maximum normal impact force. Both linear sliders (6, 11) featured low friction coefficient and excellent repeatability. All data from both directions were received by an acquisition card and then sent to the corresponding application software in a computer.

To analyze wear resistance of three different test samples, wear conditions under different numbers of impact cycles were investigated. Table 1 provides the details of experimental parameters. Each test was repeated thrice to ensure reliability of results. To well observe wear condition of each test sample, silicon nitride ceramic ball (Φ 9.52 mm) was used as impact head, as this material possesses relatively high hardness and elastic modulus. 

In this test, both x and z direction motors were oscillated with a sinusoidal wave, and the phase difference for both directions was π/2. Equations (1) and (2) show the displacement equations of both directions in each single impact-sliding cycle.

(1) x=100sin8πt

(2) z=200sin(8πt+π/2)

**Figure 3:**
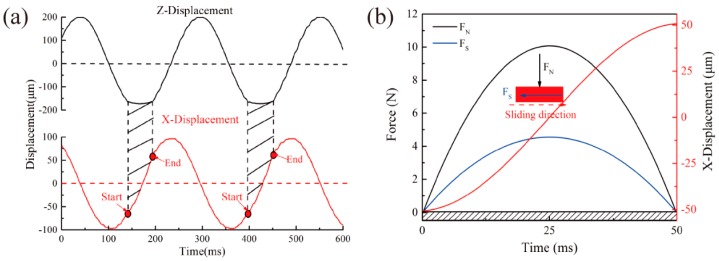
Movement and force conditions of both directions during each impact-sliding wear cycle: (**a**) Independent trajectory of each direction; (**b**) F-t diagram of each cycle.

Figure 3 displays movement, force conditions of impact head, and the test sample in each impact-sliding wear cycle. Vibration frequency of both directions are all 4 Hz. Thus, only single one-way fretting wear occurred during each impact-sliding wear cycle (Figure 3a). Maximum impact load reached 10 N, and motion status of both directions was maintained in a constant state. During each wear cycle, each sample simultaneously experienced a normal impact force and a tangential force, and the sliding force always lower than the impact force (Figure 3b). 

After the test, morphology of all wear scars was observed by scanning election microscopy (JSM6610LV). Profiles of wear scares and wear volumes were measured by white-light interferometer (Contour GT, USA).

## 3. Results 

### 3.1. Characteristics of Materials

**Figure 4:**
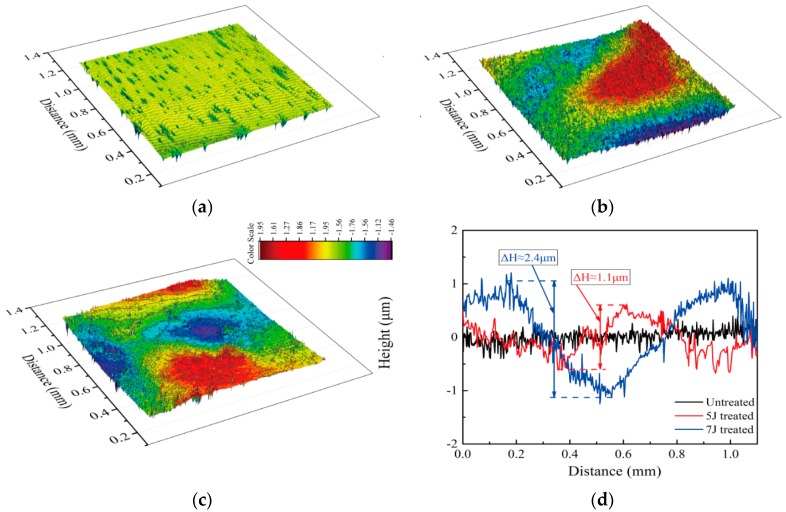
Surface morphologies of three samples. (**a**) Untreated; (**b**) 5 J treated; (**c**) 7 J treated; (**d**) 2D surface topography of three samples.

Figure 4 compares the surface topography, measured by a Nano Map-Dual Mode 3-D Profilometer, of the test samples. Altitude variation of the original sample was about 0.2, but it increased to 1.1 and 2.4 μm after the sample was peened by 5 J and 7 J pulse energies, respectively. Surface plastic deformation mainly resulted from the spatially non-uniform Gaussian distribution of shock pressure. The co-existing α and β phases with different microstructures and mechanical properties also influenced surface condition after LSP [24]. 

**Figure 5:**
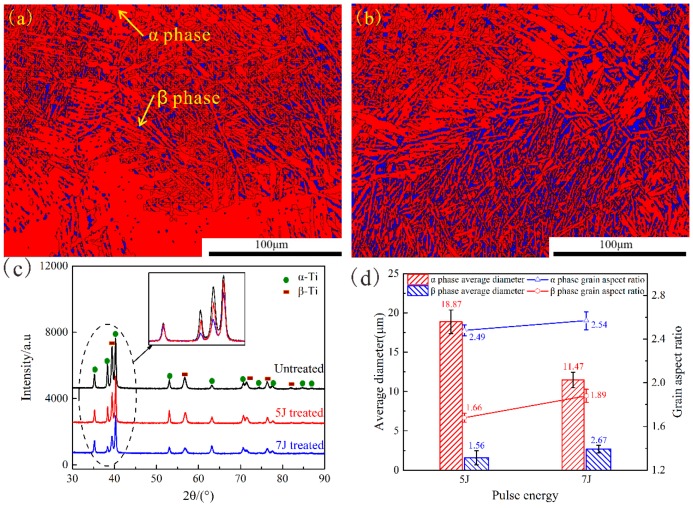
The electron backscatter diffraction (EBSD) phase maps for 5 J (**a**) and 7 J (**b**) treated samples, X-ray diffraction (XRD) patterns of three test samples (**c**); and average diameter and grain aspect ratio of both treated samples (**d**).

The electron backscatter diffraction (EBSD) technique was used to analyze microstructure changes in both treated samples, and data analysis was carried out by using the HKL-Channel 5 software. Figure 5a,b how the phase maps of the treated samples, the α phase is colored with red, whereas the β phase is marked with blue. Only α and β phases were detected in treated samples, and both displayed random orientations. The dispersed β phase was more visible in the 7 J-treated sample than in the 5 J-treated sample. When laser pulse energy was 7 J, dislocation density increased significantly, inhibiting β→α martensitic transformation. Figure 5c shows the X-ray diffraction (XRD) patterns of these three test samples. No additional peak was observed after the occurrence of different LSP impacts; this result further proved that no phase change occurred after LSP. The peaks broadened and became smooth after LSP due to the plastic deformation caused by LSP-induced high pressure shock wave, resulting in grain refinement. As shown in Figure 5d, higher pulse energy results in more notable effects. 

**Figure 6:**
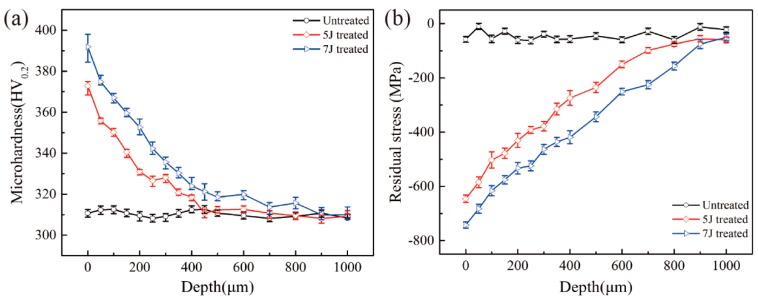
Micro-hardness (**a**) and residual stress distribution (**b**) of the three test samples.

Figure 6a shows the micro-hardness, which was measured using a micro-hardness tester, at different depths from the topmost surface of three test samples. Surface hardness of the untreated sample approximated 310 HV_0.2_, and micro-hardness values of LSP-treated surface with pulse energies of 5 J and 7 J reached 370 HV_0.2_ and 390 HV_0.2_, respectively. However, these values gradually decreased with the depth of the micro dent to the value of the untreated region. Figure 6b illustrates residual stress distribution curves on the cross-section of three test samples. All values were measured by Proto-LXRD XRD techniques, and the sample was removed layer by layer via electrolytic polishing process by which compressive residual stresses with various depths were obtained. Results showed that significant compressive residual stresses were introduced after LSP, and the maximum was constantly obtained from the material surface. Surface residual stresses of 5 J and 7 J peened samples are about 647 MPa and 750 MPa, respectively. After LSP, micro-hardness and compress residual stress of both treated samples were effectively increased, and higher pulse energy indicated clearer effects. For most metals, plastic deformation can enhance micro-hardness of the original material due to existence of high-density of dislocations in the crystals after peening [25]. Compressive stresses developed under the combinatorial effects of plastic deformation and volume limitation.

### 3.2. Wear Analysis

During the wear process, the maximum normal impact force that the samples experienced was maintained at 10 N. On the other hand, tangential force constantly changed owing to the influence of various factors, such as sample characteristic, wear conditions, and number of impact cycles. Friction coefficient was used to describe the ratio relationship between maximum impact force (F_Nmax_) and tangential sliding force (F_smax_) experienced by test samples in each impact-sliding cycle.

**Figure 7:**
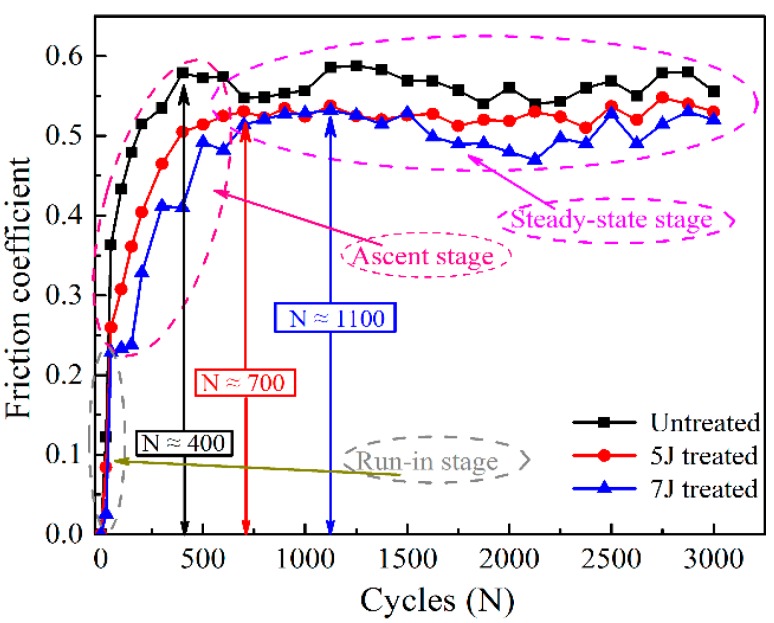
Friction coefficients among wear processes.

Figure 7 exhibits the changes in friction coefficient of different test samples with increasing impact cycles. Changes in each test sample’s friction coefficient with increasing number of impact cycles can divided into three stages: run-in stage, ascent stage and steady-stage stage. In the first stage, the distinction between friction coefficients of each sample was non-significant. The increase in friction coefficients resulted from metal-to-metal contact and material deformation in the wear zone. After about 400, 700, and 1100 cycles, the coefficients of untreated, 5 J treated and 7 J treated sample reached the peak stage, respectively. Afterward, all values fluctuated within a small range. LSP increased the unevenness and hardness of sample surface. During the whole process of fretting wear, the 7 J treated sample always exhibited the lowest friction coefficient, whereas the untreated sample showed the highest. Given the increasing impact cycles, the effects of moving debris can reduce the friction coefficient.

**Figure 8:**
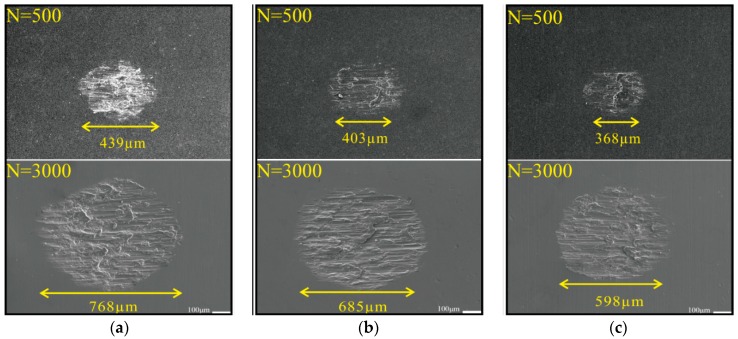
Micrographs of worn scars. (**a**) Untreated; (**b**) 5 J treated; (**c**) 7 J treated.

Figure 8 shows the micrographs of the worn scars of the three test samples under different numbers of impact cycles. All worn scars featured an elliptical shape. When wear cycles totaled 500, length values of the worn scars of untreated, 5 J treated and 7 J treated samples reached 439, 406, 368 μm, respectively. When the number of cycles increased to 3000, length values of the untreated, 5 J treated and 7 J treated samples measured about 768, 685, and 598 μm. The test sample peened by 7 J pulse energy showed the minimum wear area, whereas the untreated sample presented the largest. After treatment, wear resistance of TC17 titanium alloy improved.

**Figure 9:**
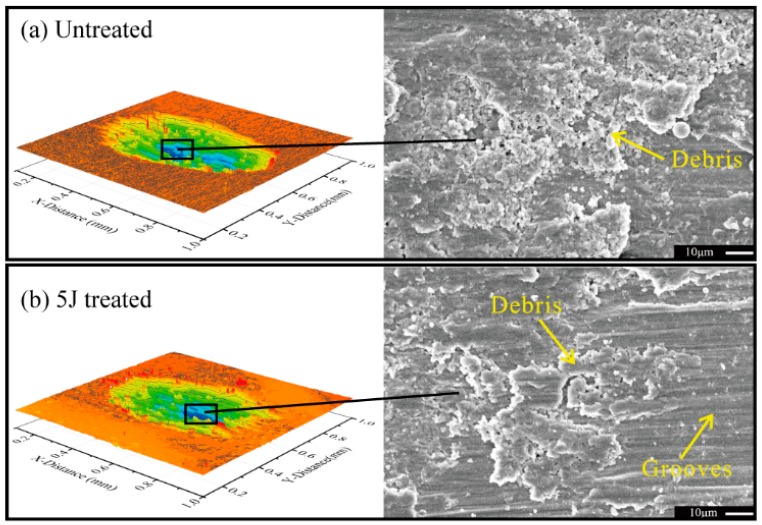

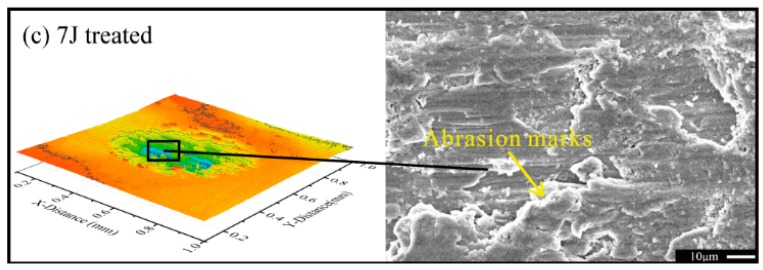
Microstructures of worn scars: N = 3000. (**a**) Untreated; (**b**) 5 J treated; (**c**) 7 J treated.

Figure 9 displays the micrographs of the middle part of the worn scars of the three test samples suffered to 3000 impact cycles. Considerable ploughing grooves were observed in the sliding direction. Some wear debris adhered to the worn surfaces, and wear debris of the untreated sample were more remarkable than those of the other two treated samples (Figure 9a). Some abrasion marks on the worn surfaces were also observed (Figure 9b), and these marks were probably induced by the abrasive wear attributed to plastic deformation of friction surfaces. Rigney reported that metal materials can produce large plastic shear strains and strain gradients, which lie at the sliding interface, and in the near surface material during sliding motions, respectively [26]. 

**Figure 10:**
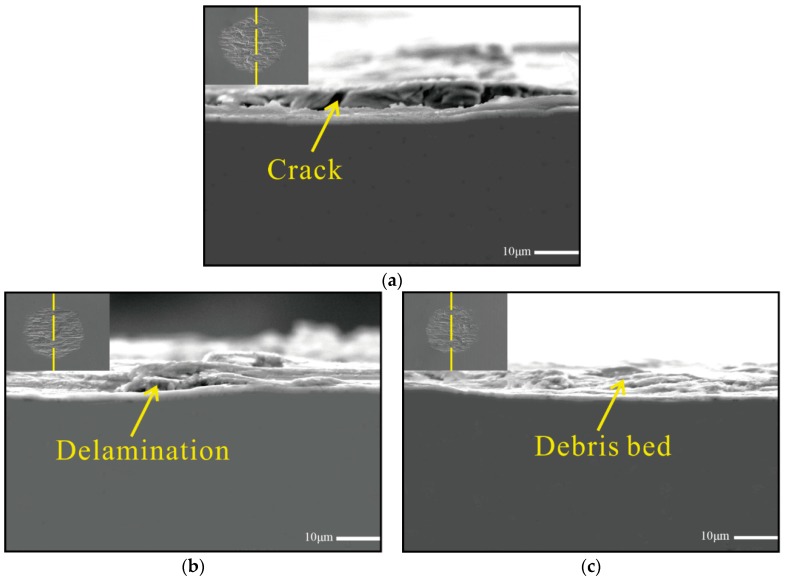
Cross-section morphology of worn scars: N = 3000. (**a**) Untreated; (**b**) 5 J treated; (**c**) 7 J treated.

Figure 10 shows cross-sections of the worn scars of each specimen. Delamination cracks were caused by fatigue effect as can be clearly observed on the untreated sample, these cracks eventually led to detachment and removal of materials. A few small shallow delaminations occurred on the surface of the test sample treated by 5 J pulse energy, whereas delamination almost wasn’t clearly shaped in the 7 J shocked sample. These findings indicated that compressive residual stress has been introduced after LSP, and this kind stress can extend the fatigue life of the metal components. In addition, residual stress cannot only impede the initiation of fatigue delamination cracks, but also reduce the extension speed of fatigue delamination, and the higher pulse energy cause more distinct effects.

**Figure 11:**
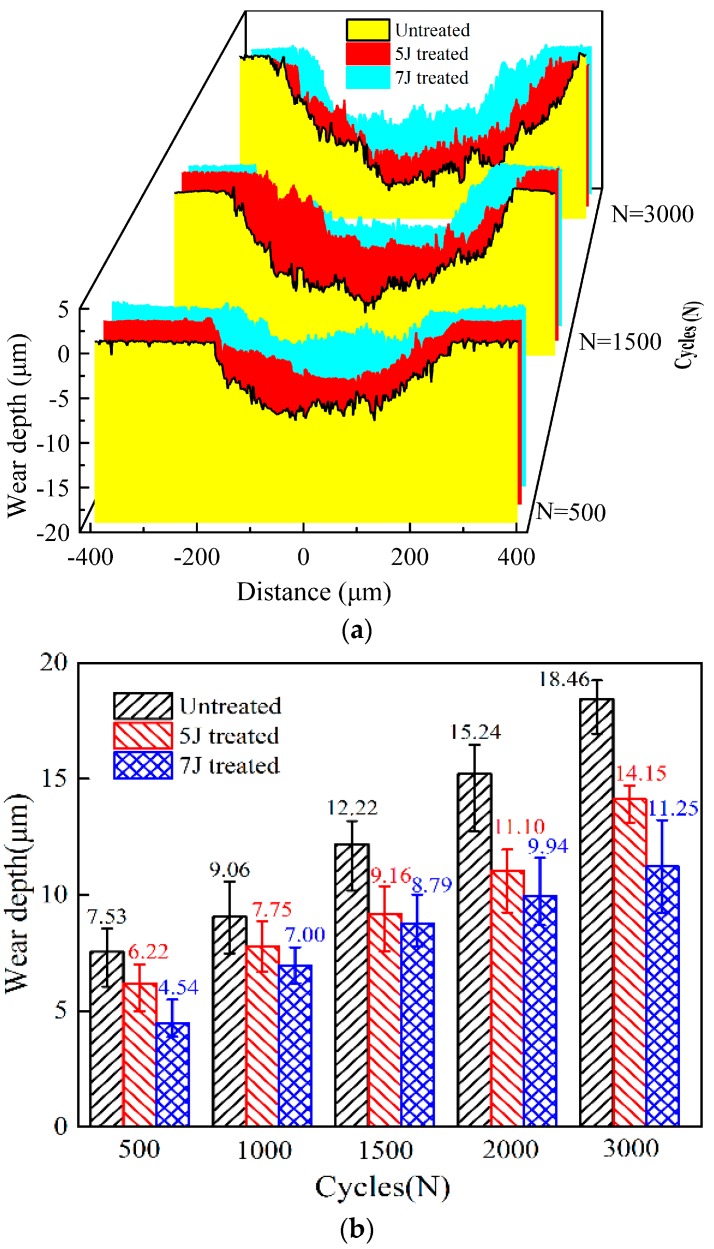
Profile of wear scars under various number of cycles. (**a**) 2-D Profile of wear scars; **(b**) Maximum wear depth.

Figure 11 presents the wear depth of the wear scars of the three test samples under different wear cycles. After 500 cycles of impact-sliding fretting wear, maximum wear depth of untreated, 5 J and 7 J treated test samples totaled about 4.54, 6.22, and 7.53 μm, respectively. When wear cycles of the samples amounted to 3000, all values reached about 11.25, 14.15, and 18.46 μm, respectively. Maximum wear depth of each test sample increased with increasing wear cycles. Maximum wear depth of the 7 J treated test sample was the smallest in the same impact cycles, but that of untreated sample was the highest mainly because surface hardness and residual compressive stress of both treated samples increased. 

**Figure 12:**
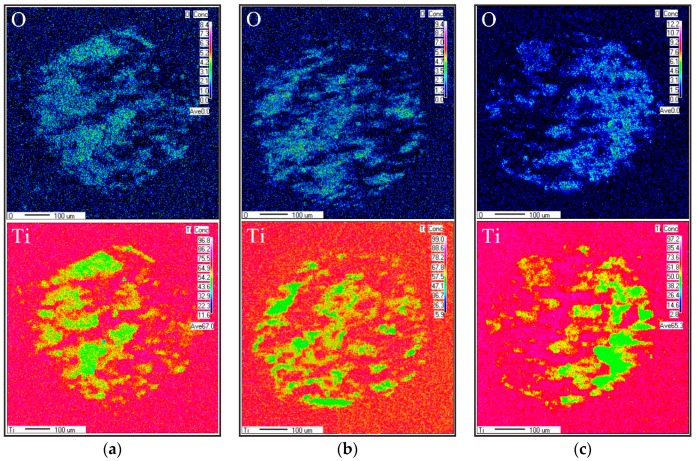
Electron probe microanalysis (EPMA) analysis on worn surfaces of three test samples, N = 3000. (**a**) Untreated; (**b**) 5 J treated; (**c**) 7 J treated.

Figure 12 shows the electron probe microanalysis (EPMA) distribution mappings of O and Ti on the worn scars of each test sample. Contents of O and Ti were measured due to the accumulated wear debris. Higher debris content in fretted area indicates higher element contents. After testing, O increased in each worn scar, indicating that each sample experienced severe oxidation wear. The sample peened by 7 J pulse energy always exhibited the lowest contents of O and Ti, suggesting the lower wear rate of this sample is lower than those of samples under the same wear condition. In each wear area, the highest O and Ti elements primarily concentrated at one end because wear condition for each sample was a one-way impact-sliding wear. 

### 3.3. Summary

In this paper, we concluded that LSP technology can improve wear resistance of TC17 titanium alloy, and to a certain degree, increased pulse energy indicates better wear resistance. In this work, the author provides detailed analysis on wear scare, wear depth, and wear volume of test samples.

**Figure 13:**
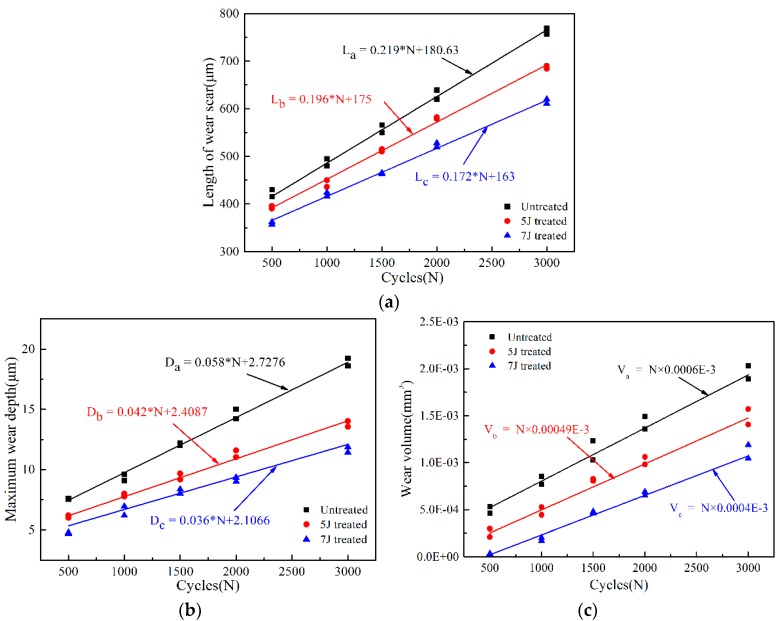
Changing rules of length, depth, and volume of the wear scars of three test samples. (**a**) Length of wear scars; (**b**) Wear depth of wear scars; (**c**) Volume of wear scars.

For impact-sliding fretting wear, the length of the wear scare is an important index for measuring wear resistance of materials. Under different impact cycles, length growth of wear scars of the three test sample were well liner-fitted, as illustrated in Figure 13a. The test sample treated with 7 J pulse energy presented the lowest growth rate, whereas the untreated sample exhibited the highest value. Growth trend of maximum wear depth of three test samples also yielded the same result, as shown in Figure 13b. Therefore LSP technology cannot only reduce but also slow down fretting wear. 

For impact-sliding fretting wear, wear volume constantly affected by normal impact load (F_N_), sliding distance (ΔS) in horizontal direction and hardness of worn surface (HV). Wear volume is proportional to impact load and sliding distance but inversely proportional to surface hardness [27]. As shown in Figure 3, the impact force and sliding distance of each test sample which suffered during each wear cycle has been basically the same. Thus wear volume of each test sample increased with increasing number of impact cycles. The sample treated by 7 J pulse energy obtained the highest surface hardness. Hence, this sample featured a minimum wear volume (Figure 13c).

## 4. Conclusions

(a) LSP generated microdents on the surface of TC17 titanium alloy which induced plastic deformation and residual compressive stress on this material. The improvement in the impact-sliding wear resistance of TC17 titanium alloy after LSP was caused by the increased surface hardness and compressive residual stress.
(b) Under the same impact-sliding wear condition, the test sample treated by 7J pulse energy exhibited the best wear resistance, whereas the untreated sample showed the poorest results. As impact-sliding cycles increased, growth rates of wear area, wear depth, and wear volume of the three test samples varied. Therefore, lower growth rate was obtained when pulse energy was higher. The main wear mechanisms of the three test samples included delamination and abrasive wear.

## Figures and Tables

**Table 1:** Experimental conditions for the impact-sliding fretting wear test.

**Number of cycles (N)**	500, 1000, 1500, 2000, 3000
**Maximum impact force (N)**	10
**Amplitude in x-direction (μm)**	100
**Amplitude in z-direction (μm)**	200
**Frequency (Hz)**	x: 4, z: 4
**Temperature (°C)**	25
**Number of experiments**	3
